# Use of Digital Technology to Enhance Tuberculosis Control: Scoping Review

**DOI:** 10.2196/15727

**Published:** 2020-02-13

**Authors:** Yejin Lee, Mario C Raviglione, Antoine Flahault

**Affiliations:** 1 Institute of Global Health, Faculty of Medicine University of Geneva Geneva Switzerland; 2 Global Studies Institute University of Geneva Geneva Switzerland; 3 Centre for Multidisciplinary Research in Health Science (MACH) Università di Milano Milan Italy

**Keywords:** tuberculosis, mHealth, eHealth, medical informatics

## Abstract

**Background:**

Tuberculosis (TB) is the leading cause of death from a single infectious agent, with around 1.5 million deaths reported in 2018, and is a major contributor to suffering worldwide, with an estimated 10 million new cases every year. In the context of the World Health Organization’s End TB strategy and the quest for digital innovations, there is a need to understand what is happening around the world regarding research into the use of digital technology for better TB care and control.

**Objective:**

The purpose of this scoping review was to summarize the state of research on the use of digital technology to enhance TB care and control. This study provides an overview of publications covering this subject and answers 3 main questions: (1) to what extent has the issue been addressed in the scientific literature between January 2016 and March 2019, (2) which countries have been investing in research in this field, and (3) what digital technologies were used?

**Methods:**

A Web-based search was conducted on PubMed and Web of Science. Studies that describe the use of digital technology with specific reference to keywords such as TB, digital health, eHealth, and mHealth were included. Data from selected studies were synthesized into 4 functions using narrative and graphical methods. Such digital health interventions were categorized based on 2 classifications, one by function and the other by targeted user.

**Results:**

A total of 145 relevant studies were identified out of the 1005 published between January 2016 and March 2019. Overall, 72.4% (105/145) of the research focused on patient care and 20.7% (30/145) on surveillance and monitoring. Other programmatic functions 4.8% (7/145) and electronic learning 2.1% (3/145) were less frequently studied. Most digital health technologies used for patient care included primarily diagnostic 59.4% (63/106) and treatment adherence tools 40.6% (43/106). On the basis of the second type of classification, 107 studies targeted health care providers (107/145, 73.8%), 20 studies targeted clients (20/145, 13.8%), 17 dealt with data services (17/145, 11.7%), and 1 study was on the health system or resource management. The first authors’ affiliations were mainly from 3 countries: the United States (30/145 studies, 20.7%), China (20/145 studies, 13.8%), and India (17/145 studies, 11.7%). The researchers from the United States conducted their research both domestically and abroad, whereas researchers from China and India conducted all studies domestically.

**Conclusions:**

The majority of research conducted between January 2016 and March 2019 on digital interventions for TB focused on diagnostic tools and treatment adherence technologies, such as video-observed therapy and SMS. Only a few studies addressed interventions for data services and health system or resource management.

## Introduction

### Background

Tuberculosis (TB) is among the top 10 causes of death worldwide, the leading cause from a single infectious agent, above HIV or AIDS, and the leading killer of people with HIV [1]. The most vulnerable people are the poorest, with 95% of cases and 98% of deaths occurring in low- and middle-income countries [2]. Although most TB deaths are preventable if detected and treated at an early stage, TB still caused an estimated 1.5 million deaths in 2018 [1].

In September 2015, the Global TB Program of the World Health Organization (WHO) developed an agenda for action on digital health exploring what contributions can be offered by this technology to the care and control of TB. This agenda highlighted opportunities and the latest information available on the use of digital health technology to combat TB [3]. Its use was categorized into 4 types of function. First, patient care and electronic directly observed therapy (eDOT), mainly refer to TB screening, TB diagnosis, and treatment adherence. As part of the latter, eDOT concerns the general recommendation of supervising and supporting patients when they take their TB drugs, thus ensuring the regular intake of medicines at home and the avoidance of daily or frequent visits to clinics. Second, surveillance and monitoring covering health information system management, measurement of the burden of TB disease and death, and the monitoring of drug resistance. Third, program management includes items such as drug stock management systems, the development of norms, and training. Fourth, electronic learning (e-learning) is the function by which electronic media and devices are used as tools for improving access to training, communication, and interaction [4].

Previously, directly observed therapy was the standard of care to ensure treatment adherence by patients throughout their long treatment duration and monitoring for adverse drug effects [5]. However, ensuring patients’ adherence to the full course of medications has traditionally been a critical challenge in TB treatment as patients needed to be observed by a health provider in a health facility, or the health provider, including community workers, had to visit the patients daily. After the introduction of digital health technology, eDOT became a significant part of digital health interventions (DHIs). Many studies were conducted around video-observed therapy (VOT), SMS, and mobile apps. In 2010, the GeneXpert *Mycobacterium tuberculosis* (MTB)/rifampicin (RIF) assay was introduced, after which an increasing number of studies assessed digital health technology in the identification of active TB cases. Most high-income countries use digital diagnostic tools to reduce diagnostic delays and prevent further transmission in the community [6].

In 2018, the WHO released a general classification on DHIs that are applicable to all conditions [7]. This classification is organized by the targeted primary user: clients, health care providers, health systems or resource managers, and data services. First, clients are the potential or current users of health services. Second, health care providers are members of the health workforce who deliver health services. Third, health system managers and resource managers are involved in administrative or surveillance works, including supply chain management, health financing, and human resource management. Finally, data services consist of supporting a wide range of activities related to data collection, management, use, and exchange.

### Objective

To achieve the End TB Strategy milestones for 2020 and 2025—*TB incidence needs to be falling by 10% per year by 2025, and the proportion of people with TB who die from the disease needs to fall to 6.5% by 2025*—as well as the 2030 to 2035 global targets, digital health is considered critical [3]. In other words, the existing approaches to patient care, surveillance and monitoring, program management, and e-learning could be strengthened by the utilization of digital health technologies, including mobile phones, big data, genetic algorithms, and artificial intelligence.

The goal of this scoping review was to provide an overview of the publications covering this subject. The results of this study could ultimately be applied to enhance the use of digital technology in TB control more sustainably and effectively. This study answers 3 main questions. First, to what extent has the subject been covered in the scientific literature between January 2016 and March 2019? Second, which countries were investing in research in this field? Finally, what digital technologies were used? The study compares results based on 2 types of classifications: one by function and the other by targeted user.

## Methods

### Scoping Review 

A scoping review documents the entire process in sufficient detail, which could be replicated by other scholars (Textbox 1). It assigns a more precise meaning to ambiguous terms and includes them in search criteria, which makes this review evidence based. In addition, a scoping review excludes the quality of papers from the selection criteria, meaning that it is less biased in the inclusion criteria [8].

**Five processes of scoping review.**

1. Identify the research question with a broad approach.
2. Identify relevant studies.
3. Study selection.
4. Chart the data by synthesizing and interpreting the qualitative data.
5. Collate, summarize, and report the results.

Following the framework of a standard scoping review, this work first identified research questions that were as wide as possible to include all of the relevant studies on the use of digital health technology for TB care and control. Afterwards, relevant studies were collected from 2 major databases pertinent to global health, followed by the process of study selection. Finally, the findings were categorized into 4 types of interventions following logic derived from 2 WHO-recommended approaches. One by function, including patient care, surveillance and monitoring, program management, and e-learning [4], and the other by the primary targeted user, such as clients, health care providers, health system or resource managers, and data services [5].

### Search Strategy

To identify all relevant studies, a comprehensive search strategy was developed to include, but not be confined to, (*tuberculosis* OR *tuberculosis infection* OR *TB* OR *tuberculosis disease* OR *mycobacterium tuberculosis*) AND (*digital* OR *ehealth* OR *mhealth* OR *technology* OR *telemedicine* OR *mobile* OR *big data* OR *artificial intelligence* OR *real-time* OR *video*). These search terms were used to identify relevant literature in 2 primary databases, PubMed and Web of Science.

### Study Selection

The scoping review included articles covering both quantitative and qualitative research, systematic reviews, editorials and viewpoints, and correspondence indexed in the PubMed or Web of Science databases. The publication dates ranged from January 2016 to March 2019. This date range was selected to cover the period after the WHO recommended date for worldwide adoption of the new End TB Strategy in 2016. On the basis of the inclusion and exclusion criteria, the articles collected were screened for relevance. The first selection was made by reviewing the titles and abstracts of the articles. English, Chinese, and French languages were considered for selection, whereas Russian was excluded. A final selection was made after reviewing the full texts.

**Figure 1:**
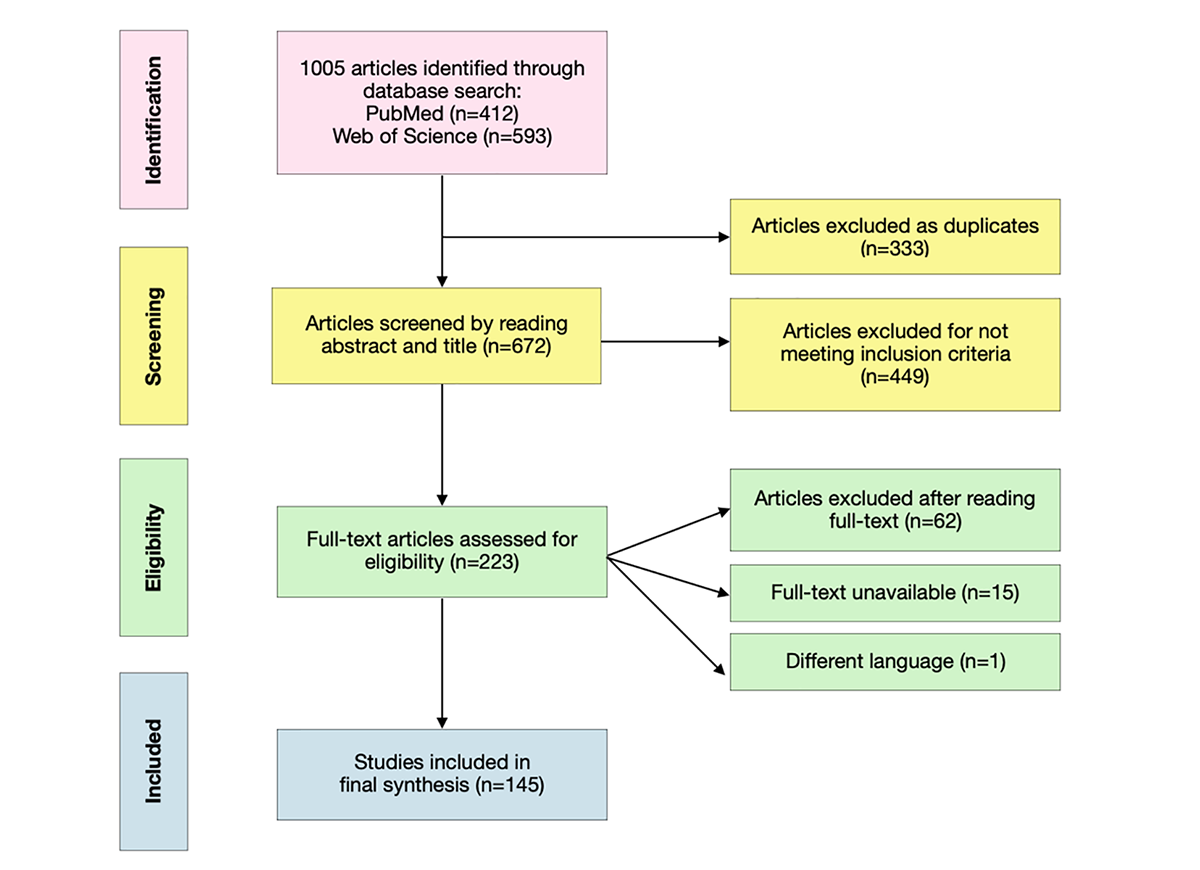
Flowchart of literature search and screening.

Of the original 1005 articles, 333 were excluded as duplicate studies, and 449 did not meet the inclusion criteria based on the title and abstract. As a result, 223 articles were assessed in full. Articles were eligible for inclusion if they focused on the use of digital health technologies in TB patient care, surveillance, programmatic function, or e-learning. Articles on bovine TB, TB drug development, epidemiology of TB, or evaluation on the quality of technology were excluded. After full reading of the 223 articles, 62 were excluded, and 1 article (in Russian) without English or Chinese summary was also excluded. A total of 15 articles, which were not available in full text, but for which only the conference abstracts or summary existed, were excluded. Of the original 1005, 527 studies did not meet the inclusion criteria (511 were not relevant, 15 were not available with full-text, and 1 was in Russian). Therefore, a total of 145 studies, including 140 in English and 5 in Chinese, were finally identified as relevant (see Multimedia Appendix 1 [3,6,9-150]). Figure 1 summarizes the flow of literature search and screening.

### Data Synthesis

In the analysis, a descriptive numerical summary is provided to present the following information: author/s, publication year, study type, geographic region of the study, the first author’s affiliation country, digital health technology domain, interventions of digital technology, and the main results. The geographic origin of the papers was categorized according to the World Bank regional grouping, which includes East Asia and Pacific, Latin America and Caribbean, North America, Sub-Saharan Africa, Europe and Central Asia, Middle East and North Africa, and South Asia [151]. Papers that did not focus on a specific region or country or studies on more than one region were classified as *global*. The extracted data were extrapolated into a data charting form in a Microsoft Excel file.

## Results

### Main Results

In the assessment by function, 105 studies identified the primary use of digital technology as TB patient care. This included TB diagnosis, treatment, and care support (Table 1). A total of 30 studies used digital technology in surveillance and monitoring, including electronic medical records and information systems; 7 focused on program management, and 3 focused on e-learning.

Using the other WHO classification of the use of digital technology in health by targeted user, of the 145 studies, 107 (73.8%) studies focused on health care providers, 20 (13.8%) studies targeted clients, 17 (11.7%) studies data services, and 1 (0.7%) study the health system or resource managers. The vast majority of scientific literature targeted health care providers compared with patients or general health system managers.

Using the other WHO classification of the use of digital technology in health by targeted user, of the 145 studies, 107 (73.8%) studies focused on health care providers, 20 (13.8%) studies targeted clients, 17 (11.7%) studies data services, and 1 (0.7%) study the health system or resource managers. The vast majority of scientific literature targeted health care providers compared with patients or general health system managers.

**Table 1:** Four types of interventions.

Intervention type and digital health technology		References	
**Patient care**			
	GeneXpert		[1-11]
	Chest x-ray		[12-22]
	Polymerase chain reaction		[23-31]
	Video directly observed therapy		[32-49]
	text messages		[50-60]
	Mobile phone apps		[61-70]
	Artificial intelligence		[71-73]
	Novel technologies		[74-102]
**Surveillance and monitoring**			
	Health information system webpages (eg, OUT-TB, e-TB, ETR.Net, TB portals, and TB Genova network)		[103-133]
	Program management	[134-140]	
**Electronic learning**			
	Digital platform for chest x-ray training		[141]
	Educational video		[142]
	Mobile app		[143]

### First Authors’ Affiliation

**Figure 2:**
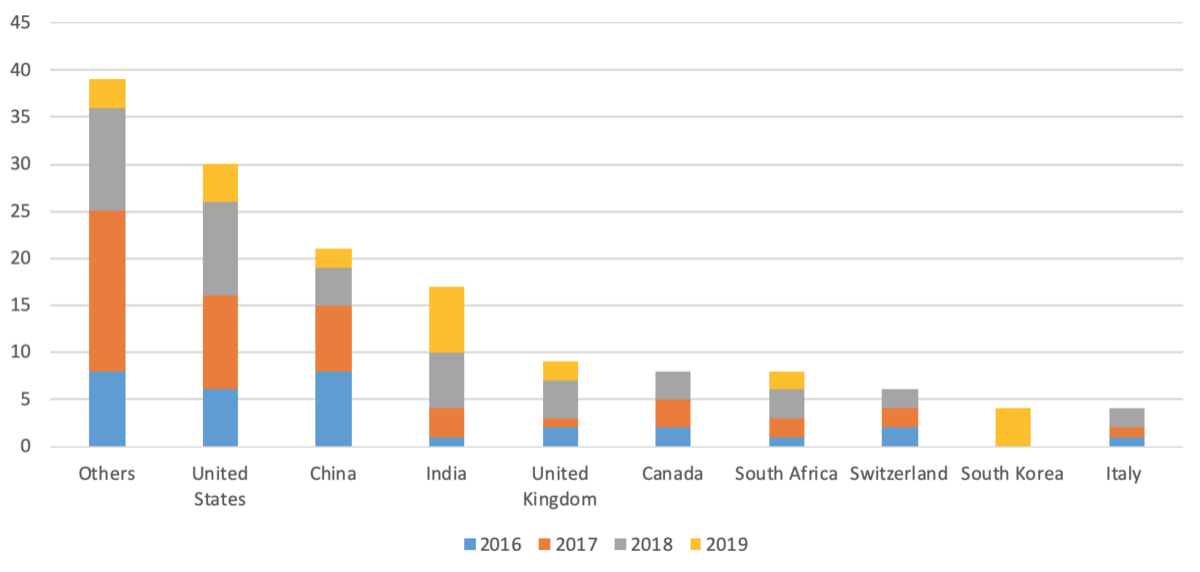
Number of publications by first author’s affiliation.

In this study, the first author’s affiliation is defined by the country of the author’s academic institution rather than the nationality of the author. The first author’s affiliation included both high- and low-income countries. In terms of frequency of publications, the following countries were identified: the United States, China, India, the United Kingdom), Canada, South Africa, Switzerland, South Korea, and Italy. Figure 2 shows that the United States was the country with the highest number of publications on this topic. Out of the 30 studies published in the United States, 11 had a geographic focus on regions outside of North America, including Sub-Saharan Africa, Latin America, and Caribbean regions. China and India were the second and third countries in terms of the number of publications when the first author’s affiliation was used as a criterion. Considering the burden of disease, it is not unusual to see the growing interests of China and India in the use of digital health technology in TB.

### Types of Digital Technology

Of the 105 studies on patient care, 62 analyzed the use of digital technology in diagnosis and 43 its use in treatment adherence. Among the 62 studies on digital technology for diagnosis, 16 were on GeneXpert MTB/RIF, which is today considered the test of choice for early and rapid diagnosis of TB [10,11]. The other studies were on digital chest x-ray (CXR) with the computer-aided detection of TB (n=14), digital real-time polymerase chain reaction technologies (n=11), artificial intelligence (n=3), deep learning or machine learning (n=2), a dot-blot system (n=1), computational modeling (n=1), and mobile 3D-printed induration (n=1), among others (n=13).

A total of 39 studies undertook a mobile health (mHealth) approach to analyze the use of mobile phones in TB treatment adherence. This approach included VOT (n=19), SMS (n=9), mobile apps (n=6), voice calls (n=2), mobile phone 3D-printed induration (n=1), and framework studies on mHealth for TB treatment (n=2). In the 19 studies on VOT, a cost and impact analysis on VOT showed that VOT could save up to 58% of costs, in addition to alleviating inconvenience and cost when visiting the treatment center [12,13]. VOT demonstrated a promising adherence rate, which is practical and enables patients in remote areas to have easy access to treatment. The challenges of VOT lie in patient confidentiality, the management of adverse drug reactions, and technical issues [14]. Patients may be unable to read SMS messages, especially women, because of the high prevalence of illiteracy [15].

A total of 30 studies on surveillance and monitoring revealed the absence of standardized health information systems to collect data on the care and control of TB [16,17]. Digital records demonstrated fewer data quality issues than paper-based records [18] and improved patient management [19]. However, newly recruited health care workers had low confidence to use digital health technologies. To enhance national or global TB surveillance and monitoring systems, some studies (n=14/30) tested Web-based platforms, the connectivity of diagnostic technologies, and standardized health information systems. Existing systems include OUT-TB Web, e-TB Manager, ETR.Net, TB Portals, and TB Genova network. In addition, artificial neural networks, big data analysis, Web-based surveys, and mathematical modeling (10/30) were used to predict the flow of TB patients. The remaining 2 studies examined TB drug susceptibility testing based on next-generation sequencing and whole-genome sequencing.

**Figure 3:**
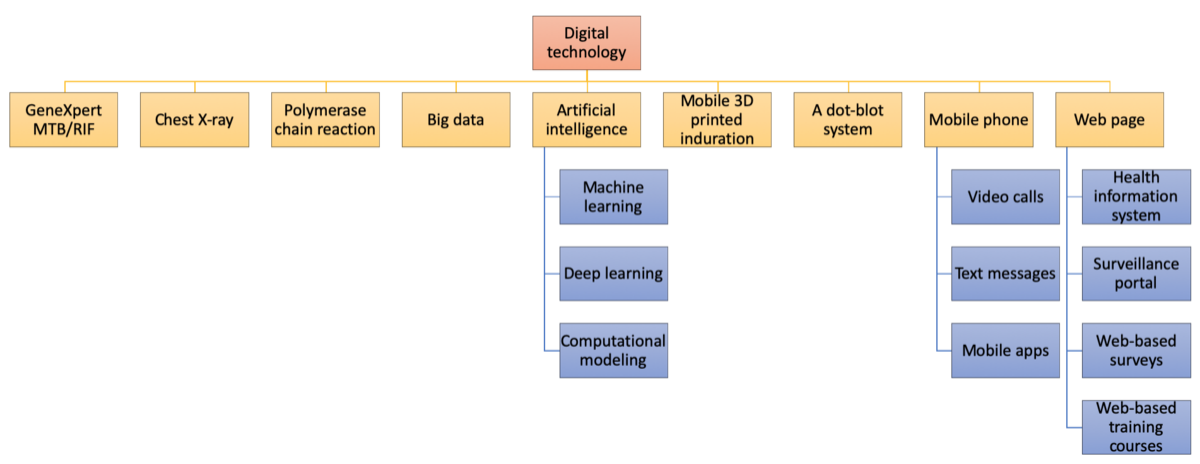
Types of digital technology. MTB: mycobacterium tuberculosis; RIF: rifampicin.

A total of 7 studies addressed the intervention of digital technology in program management. Three studies looked into the e-learning aspect of digital technology, with 1 examining a mobile phone app [20], another a Web-based training course on CXR [21], and the third a multilingual educational video on latent TB [22]. In conclusion, Figure 3 summarizes the major types of digital technology for TB that are discussed in this scoping review.

## Discussion

The findings of this scoping review suggest that the overall research efforts on the use of digital health technologies in TB care and control between January 2016 and March 2019 were focused disproportionately on patient care (105/145, 72.4%) and surveillance (30/145, 20.7%), and were aimed essentially at benefiting health care providers (107/145, 73.8% of all studies).

Only 1 study called for increased patient support focus after reviewing 24 TB-related apps in use [9]. This study argued that apps for TB patient care had minimal functionality, primarily targeted frontline health care workers, and focused on data collection. Few apps were developed for use by patients, and none were designed to support TB patients’ involvement in and management of their care. A total of 3 studies out of 145 integrated perspectives of both health care providers and patients into their analysis. These findings show a clear trend in the present literature on digital health technology for TB. It centers on feedback by health professionals, rather than TB patients, in utilizing digital health technology.

Using the TB-specific categorization by function, despite recognition of its importance, only 7 studies were devoted to program management and only 3 to e-learning. One of the 7 studies on program management developed a general framework on all priority products and concepts of digital health technologies in TB [23]. Some policy reports suggested scaling up investment in digital health to enhance TB control [152]. In the assessment of the frequency of research based on the TB-specific categorization of themes, 1 reason for the scarcity of studies on programmatic challenges could be the inclusion of TB drug management in studies outside of the TB field. This scoping review did not count studies without any keywords referring to TB; therefore, other studies, which may have referred to TB program management but without the keyword *TB* could have been overlooked. Similarly, the inclusion of gray literature, such as project reports of executive groups, could have increased the percentage of studies targeting health system managers and data services. However, this was outside the aims of this scoping review.

Another reason could be the nature of academic research papers. Standard study design in health science journals prefers interventions that are comparatively discrete and well standardized. This is the reason why most researchers prefer to focus on straightforward outcomes of interventions and on strict methodological approaches. In fact, specific diagnostic tools and VOT were the subject of a substantial number of studies, and tools such as GeneXpert MTB/RIF, VOT, and SMS were more frequently assessed under randomized controlled trial (RCT) conditions. Complex interventions such as Web-based platforms, mobile apps, e-learning, or health information systems, which go beyond testing of an individual tool, are less likely to be studied through RCTs and therefore, to be the preferred theme for a researcher.

Regarding the categorization of digital health research efforts through the lens of targeted users, a disproportionate 73.8% of studies (107/145) focused on health care providers. Some other areas, for instance health systems or resource managers, are currently not well covered by research efforts. More importantly, very few studies have focused on clients revealing the need to further explore the use of digital technology in TB care from a different and more person-centered perspective to truly identify the benefits that these tools can bring to clients.

### Multifunctionality of Digital Technology

The main results categorized the existing literature by 2 types of taxonomy. Each DHI was classified into 1 of only 4 options for the sake of simplifying the analysis. However, the possibility of overlap in technological function must be considered. In other words, some digital technologies no longer have a single function or are targeting a single user but instead have multifunctionality and can target different types of users.

For instance, for the purpose of analysis, GeneXpert MTB/RIF was considered under the category of patient care. However, at the same time, it could serve as a tool for the surveillance of drug resistance. In the past, it was impossible to connect microscopy to a database. Since 2010, however, GeneXpert has enabled the synchronization of all data into the database once the test results are available. Therefore, both health professionals and data services can obtain benefits from the use of a rapid diagnostic technology. Similarly, TB surveillance tools can be used to manage the health system. OUT-TB Web provides surveillance services such as customizable heat maps for visualizing TB and drug-resistance cases. In addition, it serves program management functions such as the allocation of financial, technical, and human resources [24]. Furthermore, reports from the ETR.Net surveillance platform were used to inform and guide resource allocation at the facilities [25].

Another good example of double targets is that of VOT. In this study, VOT was categorized depending on the primary function of the technology. It was considered an intervention for health care providers if the primary purpose was consultations between remote clients and health care providers (WHO category 2.4.1). If VOT was to ensure treatment adherence by transmitting targeted alerts and reminders, then it was considered to be a tool targeting clients (WHO category 1.1.3). The difference in the targeted user clearly shows various perspectives in understanding the functions of a single technology.

### Limitations and Direction for Further Research

This review has some limitations. One is related to the first author’s affiliation. To capture which countries invested the most in research in this field, we simplified the analysis by equating the first author’s affiliation with a country. However, the first author’s affiliation represents neither the nationality of the author nor the affiliation of the other authors if there are more. Another limitation relates to the search strategy that could be further refined. The literature search only included 2 major databases. Some articles and gray literature presented exclusively in other databases or websites could have been missed, although we suspect that they may not have had a significant impact on the findings.

Future research should fill the gaps that we unveiled, particularly in the areas of data services, health system management, and client focus. Potential research topics that have not been well investigated to date include sustainable financing of digital health technologies used for TB, surveillance of TB diagnosis equipment stocks, TB drug forecasting, and reporting on counterfeit or substandard drugs (WHO classification 3.2) [5]. In addition, it seems worth exploring the role of other e-learning tools such as the application of game techniques to education, augmented reality, and 3D learning environments.

Furthermore, not all findings in a high- or a low-resource country may apply to another country in a different situation in terms of epidemiological trends and patient populations. Thus, it is necessary to focus further on high-burden countries where digital technology has not yet been studied properly; these may include WHO-identified high-burden countries such as Angola, Bangladesh, DR Congo, Ethiopia, Kenya, Myanmar, Nigeria, and Vietnam.

### Added Value of This Study

The strengths of this review consist of the high number of studies included and the breadth of the analysis based on 2 different taxonomies of functions and targets. It summarizes the range of research activity on the use of digital technology to enhance TB control between January 2016 and March 2019. The findings highlight a need to expand knowledge and research in health system management and data services, with a view on targeting clients rather than mainly health care workers. A discussion on the multifunctionality of digital technology also provides added value in regard to different perspectives to examine various functions of a single technology.

### Conclusions

Our findings suggest that the major hubs of research on digital health for TB include the United States, as well as China and India. It is presumably because of available resources and high disease prevalence, respectively. An interesting observation derived from the study is the multifunctionality of digital technology. Unlike single-function tools in the past, an increasing number of digital health technologies carry multiple functions. Out of 145 studies, 105 (72.4%) addressed patient care as the main focus of digital health technology, and 30 (20.7%) targeted surveillance. Program management and e-learning were 2 underrepresented topics of research. Looking at the findings from a target perspective, compared with studies targeting health care providers, studies on health system managers and data services were limited as were, of particular concern, those addressing clients. Therefore, more research and development are necessary to arrive at a broader understanding of the full potential of digital technology in the TB field. We suggest that future research should focus on program management, e-learning, and surveillance, with enhanced focus on the clients, the ultimate beneficiaries, to enhance the effectiveness of care, prevention, and control of TB and contribute to its elimination.

## Appendix

Multimedia Appendix 1 Search strategy syntax for databases PubMed and Web of Science.

## Abbreviations

CXR: chest x-ray
DHI: digital health intervention
eDOT: electronic directly observed therapy
e-learning: electronic learning
mHealth: mobile health
MTB: mycobacterium tuberculosis
RCT: randomized controlled trial
RIF: rifampicin
TB: tuberculosis
VOT: video-observed therapy
WHO: World Health Organization
