# Supplementary material for: Use of Digital Technology to Enhance Tuberculosis Control: Scoping Review
Source: J Med Internet Res. 2020 Feb 13;22(2):e15727. doi: 10.2196/15727 (PMC7055857; doi:10.2196/15727)
Supplement: Multimedia Appendix 1 [file jmir_v22i2e15727_app1.docx]

## Appendix

**Search strategy syntax for databases PubMed and Web of Science**

PubMed title search term: **412 results**

Search (((tuberculosis[Title] OR tuberculosis infection[Title] OR tuberculosis disease[Title] OR TB[Title] OR mycobacterium tuberculosis[Title] OR multidrug resistant tuberculosis[Title] OR MDR TB[Title])) AND (ehealth[Title] OR mhealth[Title] OR digital[Title] OR electronic[Title] OR mobile[Title] OR technology[Title] OR telemedicine[Title] OR telemonitor[Title] OR eprescribing[Title] OR data science[Title] OR health data[Title] OR patient data[Title] OR e-patient[Title] OR big data[Title] OR mobile data[Title] OR data storage[Title] OR online[Title] OR internet[Title] OR network[Title] OR information[Title] OR application[Title] OR software[Title] OR app[Title] OR mobile device[Title] OR mobile phone[Title] OR smartphone[Title] OR cellphone[Title] OR telephone[Title] OR portable[Title] OR social media[Title] OR cloud[Title] OR web[Title] OR web-database[Title] OR mapping[Title] OR tracking[Title] OR artificial intelligence[Title] OR device[Title] OR computer[Title] OR real-time[Title] OR call[Title] OR text[Title] OR skype[Title] OR whatsapp[Title] OR video[Title])) AND ("2016.01"[Date - Publication] : "2019.03"[Date - Publication])

Web of Science advanced search term: **593 results**

Search TI=(tuberculosis OR tuberculosis infection OR tuberculosis disease OR TB OR mycobacterium tuberculosis OR multidrug resistant tuberculosis OR MDR TB) AND TI=(ehealth OR mhealth OR digital OR electronic OR mobile OR technology OR telemedicine OR telemonitor OR eprescribing OR data science OR health data OR patient data OR e-patient OR big data OR mobile data OR data storage OR online OR internet OR network OR information OR application OR software OR app OR mobile device OR mobile phone OR smartphone OR cellphone OR telephone OR portable OR social media OR cloud OR web OR web-database OR mapping OR tracking OR artificial intelligence OR device OR computer OR real-time OR call OR text OR skype OR whatsapp OR video) AND PY=(2016-2019)
